# MicroRNA miR-124-3p suppresses proliferation and epithelial–mesenchymal transition of hepatocellular carcinoma via ARRDC1 (arrestin domain containing 1)

**DOI:** 10.1080/21655979.2022.2051686

**Published:** 2022-03-18

**Authors:** Qiannan Zhao, Fen Jiang, Hui Zhuang, Yanfeng Chu, Fang Zhang, Chenghong Wang

**Affiliations:** a Department of Laboratory Medicine, Yantai Mountain Hospital, Yantai, Shandong, China; b Health Management Center, Qishan Hospital, Yantai, Shandong, China; c Central Blood Station Laboratory, Yantai, Shandong, China; d Clinical Laboratory, Jinan Central Hospital, Qilu Medical College, Shandong University, Jinan, Shandong, China

**Keywords:** microRNA, hepatocellular carcinoma, proliferation, epithelial–mesenchymal transition

## Abstract

Hepatocellular carcinoma (HCC) is responsible for high morbidity and mortality worldwide. Increasing evidence suggests that microRNAs intensively participate in HCC development and progression. In the current study, we aimed to explore the impact of miR-124-3p in the proliferation and epithelial–mesenchymal transition (EMT) of HCC. The RT-qPCR assay was employed to determine miR-124-3p expression in human HCC specimens and cell lines. Luciferase assay was used to validate the miR-124-3p target gene. Western Blot and RT-qPCR were performed to study the effects of miR-124-3p modulation on ARRDC1 (Arrestin Domain Containing 1) mRNA and protein expressions. MTT assay, wound healing assay, EdU assay, and Transwell assay were utilized to verify the impact of miR-144-3p modulation on HCC proliferation and EMT via ARRDC1. We found that MiR-124-3p expression downregulates in HCC. Overexpression of miR-124-3p reduced the HCC cell proliferation and EMT. Meanwhile, we determined that the expression of ARRDC1 is increased in HCC, and miR-124-3p directly binds the 3ʹUTR of ARRDC1 and inhibits its expression at mRNA and protein level, suggesting that miR-124-3p was capable of negatively modulating ARRDC1. Besides, cotransfection of ARRDC1-overexpression plasmid and miR-124-3p mimics increased the cell proliferation and EMT as compared to miR-124-3p mimics. Our study concluded that miR-124-3p directly binds the 3ʹUTR of ARRDC1 and exerts anti-tumorous effects by inhibiting the HCC proliferation and EMT. Therefore, miR-124-3p/ARRDC1 axis may serve as a novel therapeutic target to inhibit HCC growth and metastasis.

## Graphical Abstract

**Figure:**
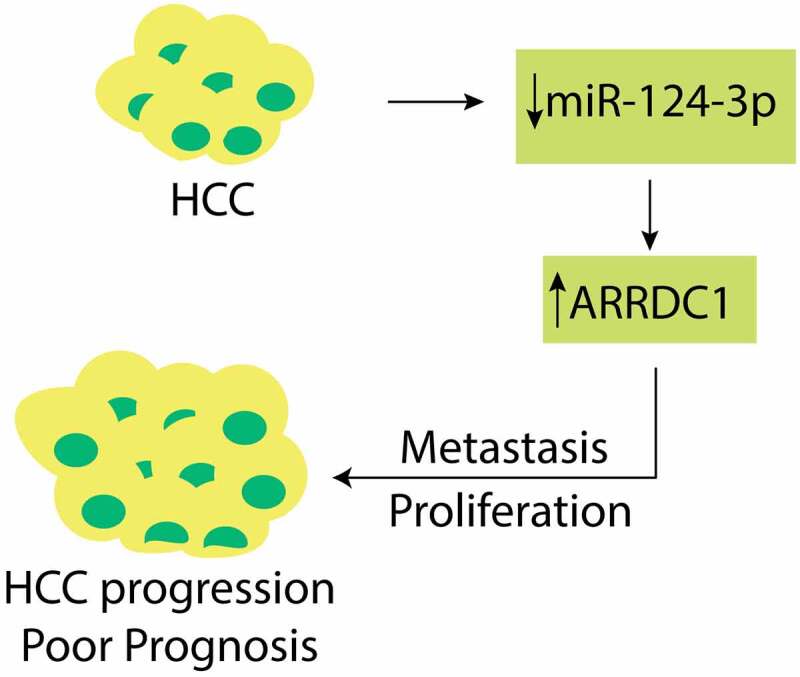

## Introduction

Hepatocellular carcinoma (HCC), regarded as end-stage liver disease, is the most common primary liver cancer, which is responsible for high morbidity and mortality across the globe [1]. Growing evidence suggests that HCC is likely to develop into the aggressive invasion and metastatic stage and spread to other organs [2,3]. So far, surgical removal of tumor and chemotherapy are considered as the only available treatment options for HCC patients. However, even though significant breakthroughs made in the past years, the prognosis is still unsatisfactory. It has been estimated that the 5-year recurrence rate of HCC after surgery reaches approximately 70% [4]. Therefore, developing a more productive and effective strategy to treat HCC is necessary.

MicroRNAs (miRNAs) are small endogenous non-coding RNAs (22 nt) [5] which participates in several biological and cellular processes, including tumorigenesis [6]. Several dysregulated miRNAs have been associated with poor prognosis among HCC patients by playing critical roles in the occurrence, progression, and development of HCC [7]. These miRNAs bind to the 3’ UTR of their target genes and regulate the cell proliferation, EMT, and metastasis of HCC [8,9]. It has been found that miR-124-3p participates in neurodegenerative diseases [10] and glioma metastasis [11]. However, the role miR-124-3p in tumors is not well understood. A recent study found that upregulation of miR-124-3p is closely associated with lower progression-free survival (PFS) among ependymoma patients [12]. Moreover, the contradictory role of miR-124-3p as an oncomiRNA and an antitumor miRNA in breast cancer has been identified [13,14]. In HCC, miR-124-3p is known to inhibit tumorigenesis through targeting ANXA7 [15]. However, it needs further investigations to understand the potential role miR-124-3p in HCC fully.

EMT is characterized by the loss of epithelial functions and acquiring mesenchymal features, which is further accompanied by degraded epithelial markers (E-cadherin) and elevated mesenchymal markers (N-cadherin, vimentin, snail) [16]. An increase in EMT contributes to stemness, tumorigenicity, metastasis, and chemoresistance [17,18]. EMT is associated with inflammatory microenvironment, fibrosis, and tumor metastasis. MiRNAs, as the critical regulator of post-transcription, regulate EMT and reduce the expression of EMT markers by negatively regulating its targets [19,20]. The role of miR-124-3p in modulating HCC growth and metastasis via ANXA7 has been documented earlier [15]. Nevertheless, the function miR-124-3p in HCC cell EMT is unclear yet.

The mammalian α-arrestin family includes five arrestin domain-containing proteins (ARRDC1-5) and TXNIP that share similar domain homology with the mammalian β-arrestins [21]. TXNIP and ARRDC1-4 (but not ARRDC5) contain two C-terminal PPXY motifs that bind WW domain-containing proteins, including the HECT-domain-containing Nedd4-like E3 ubiquitin ligases [21]. However, the role of ARRDC1 in cancer, especially HCC, is not well understood. Therefore, we designed this study to understand the role of miR-124-3p in EMT by regulating the expression of ARRDC1 (Arrestin Domain Containing 1). In this study, we evaluated the expression profile of miR-124-3p, and studied the interaction between miR-124-3p and ARRDC1. Meanwhile, we modulated the expression of miR-124-3p and/or ARRDC1 to certify miR-124-3p/ARRDC1 axis in the HCC proliferation, invasion, metastasis, and EMT.

## Materials and methods

### Clinical samples

We collected 30 specimens of HCC tumors from patients who had liver section surgeries from at Yantai Mountain Hospital between August 2017 and January 2019. Patients were not reported to have any preexisting conditions or were not under any specific drug regimen. All the specimens were immediately stored at −80 liquid nitrogen after surgery. Informed consent was signed from patients and their families. The ethical committee belonging to Jinan Central Hospital approved this study (NO.2016N11034)

### Cell culture

Human HCC cell lines, SK-Hep1, MHCC-97 H, Huh-7, and normal liver cell lines, LO-2, were purchased from Chinese Academy of Sciences Cell Bank (Shanghai). Cells were incubated in DMEM medium containing 10% FBS and 1% streptomycin/penicillin in humidified incubator at 37°C with 5% CO_2_ [22].

### Cell transfection

Cells were transfected with miR-124-3p mimics (5’-UAAGGCACGCGGUGAAUGCC-3’) or miR-NC mimics (5’-ACUACUGAGUGACAGUAGA-3’), 50 nmol/ml, which were synthesized by GenePharma, Shanghai. For the functional studies of ARRDC1, ARRDC1 overexpression plasmid (ARRDC1-OE) and its empty plasmids, 2 µg, were also provided by GenePharma. Cells were transfected with respective oligonucleotides or plasmids and were incubated for 48 h for the subsequent experiments. Transfection of the oligonucleotides or plasmids was achieved by using Lipofectamine^TM^ 2000 (Invitrogen, U.S.) [23].

### qRT-PCR

Total RNA was isolated from HCC tissues and cell lines, and cDNA was synthesized with TaqMan Reverse Transcription Kit. qRT-PCR was proceeded via PrimeScript RT Master Mix (TaKaRa). U6 and GAPDH served as loading control. The sequences of the primers used in PCR were in the following: miR-124-3p, F: 5’-GCTTAAGGCACGCGG-3’ and R: 5’-GTGCAGGGTCCGAGG-3’; U6, F: 5’-CTCGCTTCGGCAGCACATATACT-3’ and R: 5’-ACGCTTCACGAATTTGCGTGT-3’; ARRDC1, F: 5’-TAGTGGAGGAGGGTTACTTCAAC-3’ and R: 5’-TCTGGGATGCTGTTCAGGTTC-3’; GAPDH, F: 5’-CCACTCCTCCACCTTTGAC-3’ and R: 5’-ACCCTGTTGCTGTAGCCA-3’. Applied Biosystems 7500 real-time PCR (Applied Biosystems, Foster City, CA, USA) was used for qRT-PCR. All the primers were bought from IDT company. Mature miR-124-3p was used for qRT-PCR. Relative expression levels of mRNA and miRNA were calculated with the 2− ΔΔCt method [24].

### Western blot

To extract the total protein, RIPA was added to the cells (Beyotime Biotechnology, Jiangsu, China). BCA assay was used to quantify the proteins. Ten percent SDS-PAGE was used to separate proteins and then transferred on PVDF membrane. After transferring, PVDF membranes were blocked in 5% skim milk for 2 hours. Afterward, the primary antibodies, such as anti-ARRDC1 (ab181758, 1:1000), anti-E-cadherin (ab40772, 1:20,000), anti-N-cadherin (ab76011, 1:15,000), anti-Vimentin (ab92547, 1:5000) were added and incubated at 4°C overnight. After 16 hours of primary antibody incubation, TBST was used to wash membranes for three times, and the respective secondary antibodies were added for 2 hours. ECL Western Blotting Substrate was utilized to expose the proteins. ImageJ was used to quantify the proteins [22].

### MTT assay

HCC cells were cultured in 24-well plates for 24-hour-culture, later were incubated with MTT. Afterward, cells were supplemented with dimethyl sulfoxide containing purple formazan crystals. The cell proliferation was determined with microplate readers at 570 nm [25].

### Wound healing assay

HCC cells seeded into 6-well plates (3 × 10^4^ cells/well) were cultured till at the confluency of 90%, later via sterile 20 μl pipette tips to make a scratch. The floating cells were cultured with DMEM medium. Subsequently, the cells were captured via inverted optical microscopes (×200) (Nikon) [26].

### EdU assay

The transfected cells were incubated with 50 µmol/L of EdU, later fixed with 4% paraformaldehyde, permeated with 0.5% Triton-X 100 and treated with Apollo488. After that, they were cultured with Hoechst 33,342. The mounted samples were captured with inverted microscopes, and EdU positive cells were calculated [27]. Images were processed with Image J to enhance the fluorescence.

### Transwell assay

HCC cells were cultured in upper chambers precoated with Matrigel, lower ones added with 10% FBS. At 24-post incubation, the invaded cells were stained with crystal violet. Those from five fields were visualized with microscopes [28].

### Luciferase assay

TargetScan (http://www.targetscan.org/vert_72/) tool was used to predict miR-124-3p ARRDC1 as target gene. The ARRDC1 3’ UTR WT and MUT with the binding sites of miR-124-3p were synthesized and provided by GenePharma, Shanghai. These sites were cloned in pmir-GLO plasmid (Promega). HCC cells were transfected with miR124-3p mimics or NC mimics and ARRDC1 3’ UTR WT or MUT for 72 h with Lipofectamine 2000. Luciferase activity was calculated with a luciferase assay kit (Promega) [13].

### Statistical analysis

The data were evaluated with GraphPad 5, represented as mean ± SD. The statistical differences were evaluated with student *t*-test as well as ANOVA for multigroup. P < 0.05 means statistical significance difference.

## Results

### Expression of miR-124-3p is lowered in HCC tissues and cells

**Figure 1.**
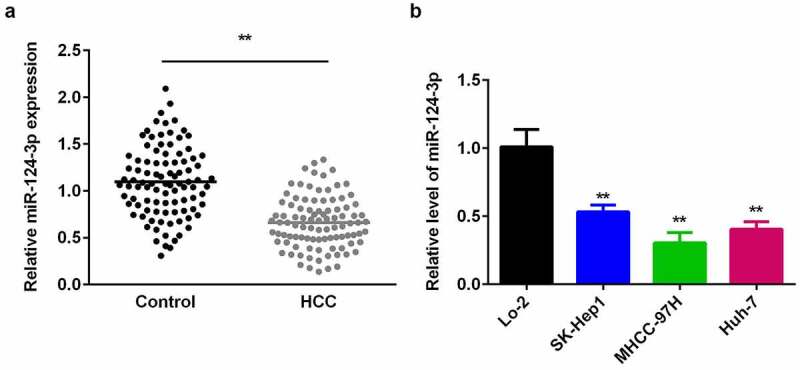
miR-124-3p is decreased in HCC tissues and cells.

Previous studies have shown that miR-124-3p association with several cancers. It has been reported that miR-124-3p play critical role in the proliferation and metastasis of HCC [15]. Therefore, we designed this study to evaluate the expression and functions of miR-124-3p in HCC. To make a preliminary explore of miR-124-3p effects on HCC, we firstly detected miR-124-3p expression in HCC cells and human tissues. First, as shown in Table 1, low-expression miR-124-3p is closely associated with lymph node metastasis and tumor stage, but with no significant difference in age and gender (Table 1). There was no statistical difference between the age (>0.127) and gender (>0.450) of patients with low or high miR-124-3p expression in human HCC patients. However, the tumor stage (>0.127) and distant metastasis (>0.450) of patients with low or high miR-124-3p expression in human HCC patients showed a statistical difference. Then, we probed to investigate the miR-124-3p expression in HCC by using qRT-PCR. Our results showed that miR-124-3p was downregulated in HCC tissues isolated from the humans (Figure 1a). Similarly, miR-124-3p was signally lowered in HCC cells (Figure 1b), which was more potent in MHCC-97 H cells. Therefore, MHCC-97 H cells were used in the following experiments.

**Table 1.** Clinical features

**Variables**	**miR-124-3p expression**		***P* values**
	Low	High	
**Age**			>0.127
**<50**	6	8	
**» 50**	5	11	
**Gender**			>0.450
**Male**	9	12	
**Female**	2	7	
**Tumor stage**			<0.029*
**I–II**	6	9	
**III–IV**	5	10	
**Distant metastasis**			<0.008*
**Yes**	4	12	
**No**	7	7	

*t*-test; *P < 0.05 means statistical significance.

A: miR-124-3p was decreased in HCC tissues. qRT-PCR detected expression level. B: miR-124-3p was downregulated in HCC cells. qRT-PCR detected expression level. *t*-test; **P < 0.01 vs. healthy control group or Lo-2 cells.

### Overexpression of miR-124-3p reduced the HCC proliferation

**Figure 2.**
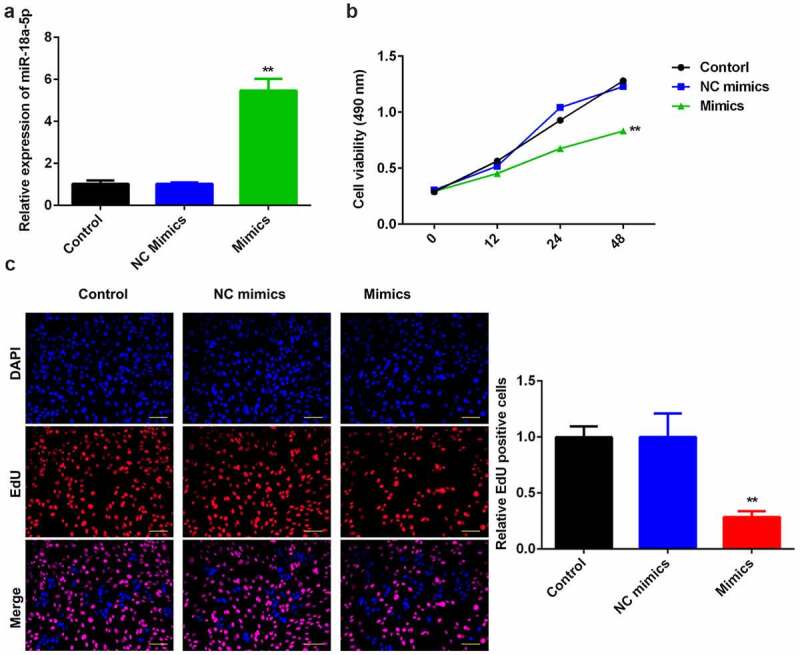
miR-124-3p modulates HCC proliferation.

To probe into the role of miR-124-3p in HCC, we used miR-124-3p mimics to evaluate its functions in HCC cell lines. We found that miR-124-3p was significantly upregulated in miR-124-3p mimics group (Figure 2a) at the dose of 50 nmol/ml, suggesting cells were successfully transfected. Later, overexpression of miR-124-3p significantly suppressed HCC cell proliferation at 24-hour and 48-hour post-transfection, as compared to control group (Figure 2b). In line with MTT assay, miR-124-3p visually decreased EdU positive cells (Figure 2c). Overall, these data indicate that miR-124-3p negatively regulated the HCC cell proliferation.

A: miR-124-3p was signally upregulated via miR-124-3p mimics. qRT-PCR determined miR-124-3p expression. B: Overexpressing miR-124-3p inhibited HCC cell viability. MTT assay measured HCC cell viability. C: Overexpressing miR-124-3p inhibited HCC cell proliferation. EdU assay detected HCC cell proliferation at (scale bar = 50 µm). *t*-test; **P < 0.01 vs. control group.

### Overexpression of miR-124-3p suppresses HCC migration and invasion ability

**Figure 3.**
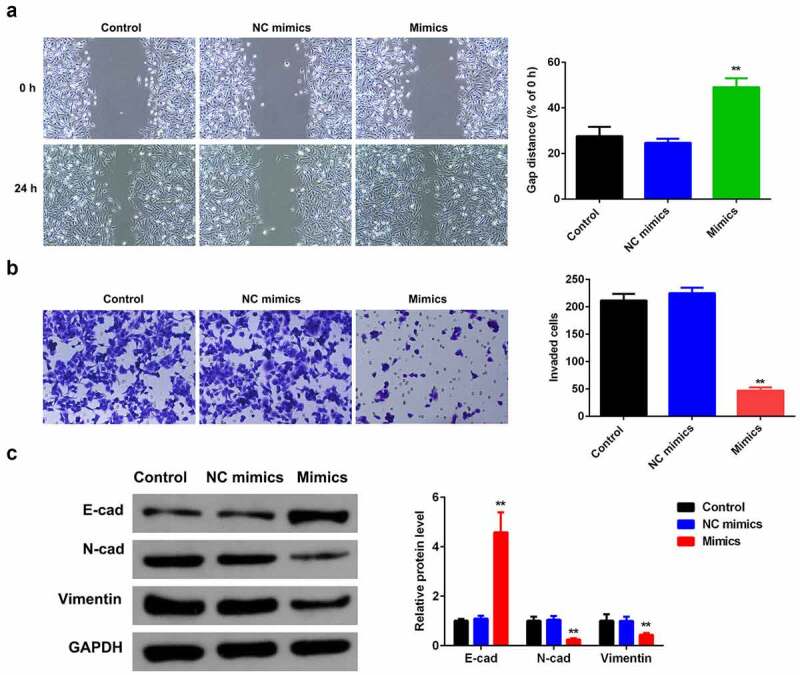
miR-124-3p suppresses HCC EMT.

To exploit the role miR-124-3p played in HCC EMT, we examined the influence of miR-124-3p overexpression on HCC cell migration and invasion ability. Overexpressing miR-124-3p significantly decreased the HCC migrated cells (Figure 3a). Meanwhile, miR-124-3p significantly lowered the number of invaded cells (Figure 3b). Furthermore, our study determined that overexpression of miR-124-3p significantly increased EMT marker E-cadherin and decreased the expression level of N-cadherin and vimentin. These findings revealed that miR-124-3p may negatively regulate HCC cells EMT.

A: Overexpression of miR-124-3p suppressed HCC cell migration ability. The wound healing assay detected HCC cell migration ability (scale bar = 50 µm). B: Overexpressing miR-124-3p inhibited HCC cell invasion ability as detected by Transwell assay (scale bar = 20 µm). C: Overexpression of miR-124-3p modulated E-cadherin, N-cadherin, and Vimentin protein levels as detected via western blotting. *t*-test; **P < 0.01 vs. control group.

### MiR-124-3p negatively regulates the expression of APPDC1 by binding 3’ UTR

**Figure 4.**
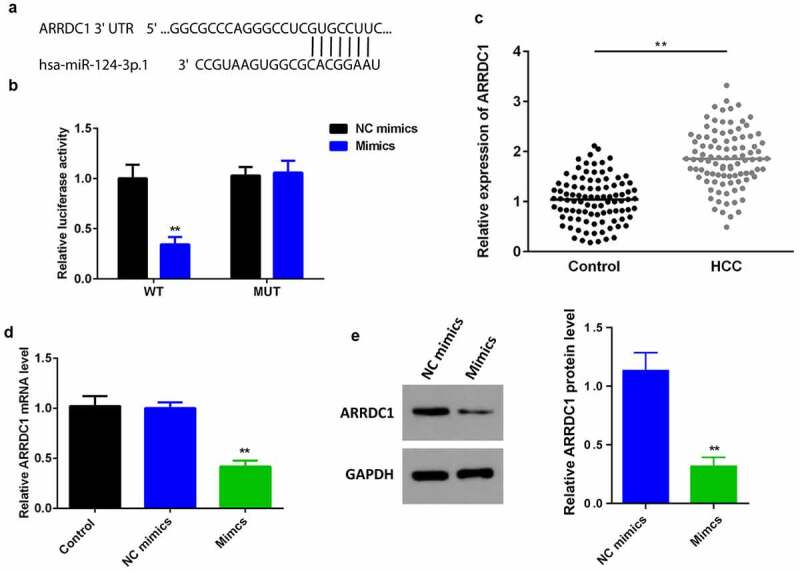
miR-124-3p directly targets ARRDC1.

It has been studied miRNAs intensively participate in the initiation and development of cancer via binding to 3’ UTR of their target genes. To probe into the underlying molecular mechanisms, we explored the possible target of miR-124-3p. It was previously reported that ARRDC1 promotes HCC progression. We explored TargetScan 7.2 (http://www.targetscan.org/vert_72/) and predicted ARRDC1 as a miR-124-3p target gene. Luciferase assay showed a reduced luciferase activity after co-transfection of HCC cell line with miR-124-3p mimics and ARRDC1 3’ UTR WT; but there was no significant difference between MUT groups (Figure 4b). Moreover, we determined that ARRDC1 was signally upregulated in HCC tissues (Figure 4c). Furthermore, we determined that the overexpression of miR-124-3p significantly downregulated the expression of ARRDC1 at mRNA and (Figure 4d) and protein (Figure 4e) level, showing miR-124-3p directly targeted ARRDC1, and negatively regulated its expression.

A: TargetScan 7.2 predicted the miR-124-3p binding sites in the 3’ UTR of ARRDC1. B: Luciferase assay showing the decrease in luciferase activity in cells co-transfected with miR-124-3p mimics and ARRDC1 3’ UTR WT. C: ARRDC1 mRNA level was significantly elevated in human HCC tissues. D: qRT-PCR showing that overexpression of miR-124-3p downregulated the expression of ARRDC1 at mRNA level. E: qRT-PCR showing that overexpression of miR-124-3p downregulated the expression of ARRDC1 at mRNA level. *t*-test; **P < 0.01 vs. control group.

### MiR-124-3p suppresses HCC proliferation and EMT via regulating ARRDC1

**Figure 5.**
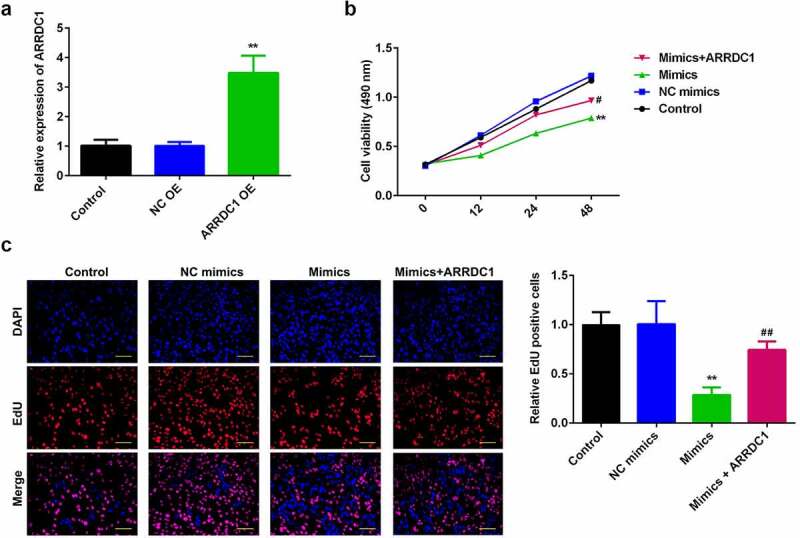
miR-124-3p suppresses HCC proliferation via regulating ARRDC1.

**Figure 6.**
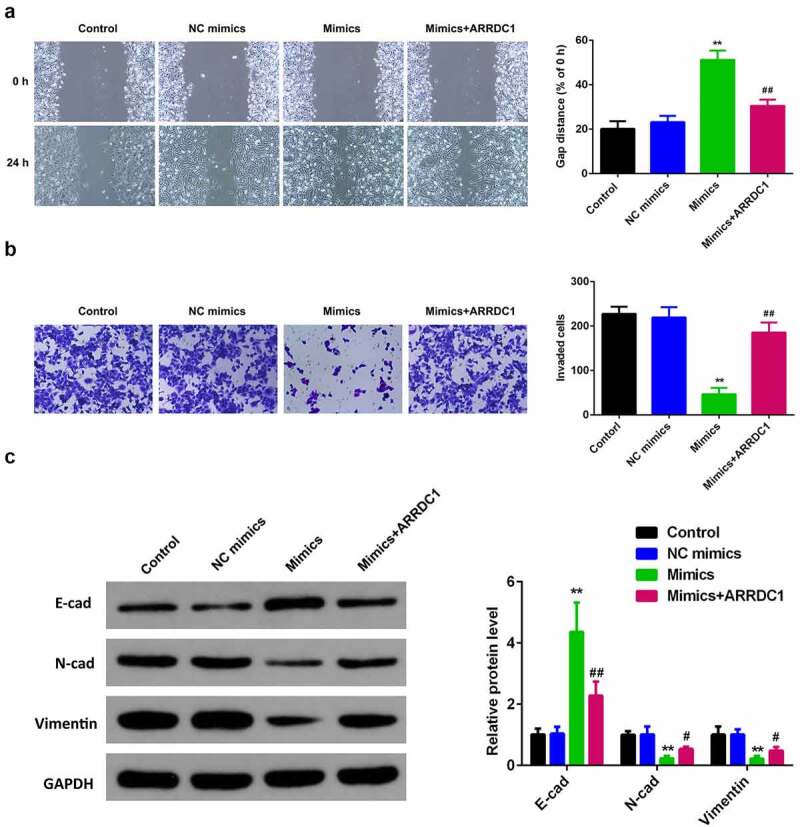
miR-124-3p suppresses EMT of HCC via regulating ARRDC1.

To further verify the role miR-124-3p played in HCC, our work performed rescue experiments. ARRDC1 expression in ARRDC1 OE group was significantly upregulated compared with control group (Figure 5a), suggesting HCC cells were successfully transfected with ARRDC1-OE plasmid. Further, we used the co-transfection of miR-124-3p with ARRDC1-OE plasmid, and detected the HCC cell proliferation. The results showed that overexpression of ARRDC1 minimized the anti-proliferative effects of miR-124-3p in HCC cells (Figure 5b-c). Moreover, miR-124-3p-induced suppression in HCC cells migration and invasion ability, but the co-transfection of miR-124-3p mimics with ARRDC1-OE plasmid significantly reversed the HCC cell migration and invasion (Figure 6a & b). Furthermore, our study determined that overexpression of miR-124-3p significantly increased EMT marker E-cadherin and decreased the expression level of N-cadherin and vimentin. However, co-transfection of miR-124-3p mimics and ARRDC1-OE decreased EMT marker E-cadherin, and increased the expression level of N-cadherin and Vimentin. Therefore, we concluded that ARRDC1 restored N-cadherin and Vimentin expressions, whereas decreased miR-124-3p-induced increase in E-cadherin (Figure 6c).

A: mRNA level was signally elevated after transfecting with ARRDC1 overexpression plasmids. qRT-PCR determined mRNA level. B: Overexpressing ARRDC1 enhanced HCC cell viability, which was determined via MTT assay. C: Overexpressing ARRDC1 promoted HCC cell proliferation. EdU assay detected HCC cell proliferation (scale bar = 50 µm). ANOVA; **P < 0.01 vs. control group. ##P < 0.01 vs. mimics group.

A: Overexpressing ARRDC1 suppressed HCC cell migration ability. The wound healing assay detected HCC cell migration ability (scale bar = 50 µm). B: Overexpressing ARRDC1 inhibited HCC cell invasion ability (scale bar = 20 µm). C: Overexpressing ARRDC1 modulated E-cadherin, N-cadherin, and Vimentin protein levels detected via western blotting. One-way ANOVA; **P < 0.01 vs. control group. ##P < 0.01 vs. mimics group.

## Discussion

HCC is characterized as one of the major reasons for cancer-related death worldwide [29]. Meanwhile, miRNAs are known to play several regulatory roles in the HCC progression and advancement [15,19,20]. Our work explored the roles of miR-124-3p in HCC proliferation and EMT. We found that miR-124-3p level was downregulated in the HCC. This data suggesting that miR-124-3p may act as a tumor suppressor in HCC. Furthermore, we explored that overexpression of miR-124-3p suppresses HCC proliferation and EMT via targeting 3’ UTR of ARRDC1.

Abnormal miRNAs expression acts as an oncomiRNA or anti-tumor miRNA [5,6,8]. miR-124-3p is reported to be an anti-tumor miRNA in human ovarian cancer, chordoma, papillary thyroid carcinoma, and HCC [15,30,31]. Similar to our study, another report also showed that dysregulation of miR-124-3p is closely associated with high tumor stage, metastasis, and poor survival in the HCC patients [15]. But overexpressing miR-124-3p represses HCC proliferation, apoptosis, and metastasis.

Clinically, HCC can be treated by surgical resection or removal of tumor. However, the chances of recurrence after surgical resection may lead to failure for treatment and badly affect the long-term survival of HCC patients [32]. Postoperative recurrent HCC can be caused by intrahepatic metastasis of the primary tumor, with a high incidence and poor prognosis [33,34]. The highly invasive properties of HCC cells into macro- and microvascular vessels can give rise to metastasis with high frequency [35]. Therefore, it is necessary to identify the HCC patients with high invasive and metastatic potential to increase the chances of curative interventions. In our study, we found that the level of miR-124-3p was robustly down-regulated in HCC with advanced stages of cancer. These findings suggest that miR-124-3p is a potential prognostic indicator for patients with HCC and can be used to predict the potential for HCC aggressive invasion, metastasis, and poor prognosis.

Metastasis is the critical factor for cancer-related death. But tumor metastasis is complex and associated with numerous pathways. Increasing evidence reveals that EMT is a classical player in the tumor metastasis theory [36]. In this study, we found that miR-124-3p suppressed the markers of EMT in the HCC cell lines. EMT process features enhanced migration and invasion, thus promoting tumor growth. miR-124-3p inhibited HCC cell migration and invasion ability. The biological EMT process is accompanied by polarity loss of epithelial cells and gradual transformation into mesenchymal phenotype, which acquires specific genetic changes, including degraded epithelial markers and elevated mesenchymal markers [^16–18^]. In this work, overexpressing miR-124-3p upregulated E-cadherin but downregulated N-cadherin and Vimentin. Existing studies reveal that EMT promotes HCC tumor growth [37]. In this study, miR-124-3p inhibited HCC proliferation. This may be a new strategy for suppressing HCC proliferation and metastasis. But the underlying molecular mechanisms are still unknown.

Augmenting evidence has revealed that miRNAs regulate HCC initiation and progression via degrading its target level [38,39]. In this study, we used the online bioinformatics tool and screened that miR-124-3p binds to the 3ʹUTR of ARRDC1. We further explored that miR-214-3p lowered ARRDC1. ARRDC1, in the mammalian α-arrestin family, functions as an adaptor for recruiting specific protein cargo into tumor-released extracellular vesicles, which, with the tumor pre-metastatic niche and metastatic potential, crucially regulates cell-to-cell communication in cancer initiation and development [40]. Moreover, ARRDC1 modulates the EMT of clear cell renal cell carcinoma via hippo pathways, whose activation, interestingly, inhibits liver cancer *in vivo* and *in vitro* [40]. Moreover, macrophage-secreted cytokines/chemokines enhance ARRDC1-induced PKM2 ectosomal release from HCC cells, which form a feedback loop for carcinogenesis, monocyte-to-macrophage differentiation, and tumor microenvironment remodeling [41]. Therefore, ARRDC1 may function as an oncogene in liver cancer and promotes the metastasis of HCC. In this study, ARRDC1 was upregulated in HCC tissues. Moreover, ARRDC1 abrogated the impact of miR-124-3p on HCC cell proliferation and restored EMT phenotype, with enhanced HCC cell migration and invasion ability. But whether miR-124-3p/ARRDC1 axis modulates HCC proliferation and EMT via functioning as a mediator for protein cargo packaged into EVs or through specific pathways needs further study.

## Conclusion

In this study, we uncovered the anti-cancerous roles of miR-124-3p. Our results indicated that expression of miR-124-3p was significantly reduced in the human HCC specimens and HCC cell lines. Moreover, miR-124-3p suppressed HCC cell proliferation and EMT by targeting the 3’ UTR and negatively regulating ARRDC1. Finally, we found that overexpression of ARRDC1 could rescue the anti-tumor effects of miR-124-3p. Overall, our study suggests that targeting miR-124-3p/ARRDC1 axis could be a promising strategy for suppressing HCC metastasis.

## Supplementary Material

Supplemental Material
