# Supplementary figures and images for: MicroRNA miR-124-3p suppresses proliferation and epithelial–mesenchymal transition of hepatocellular carcinoma via ARRDC1 (arrestin domain containing 1)
Source: Bioengineered. 2022 Mar 18;13(4):8255–65. doi: 10.1080/21655979.2022.2051686 (PMC9161870; doi:10.1080/21655979.2022.2051686)

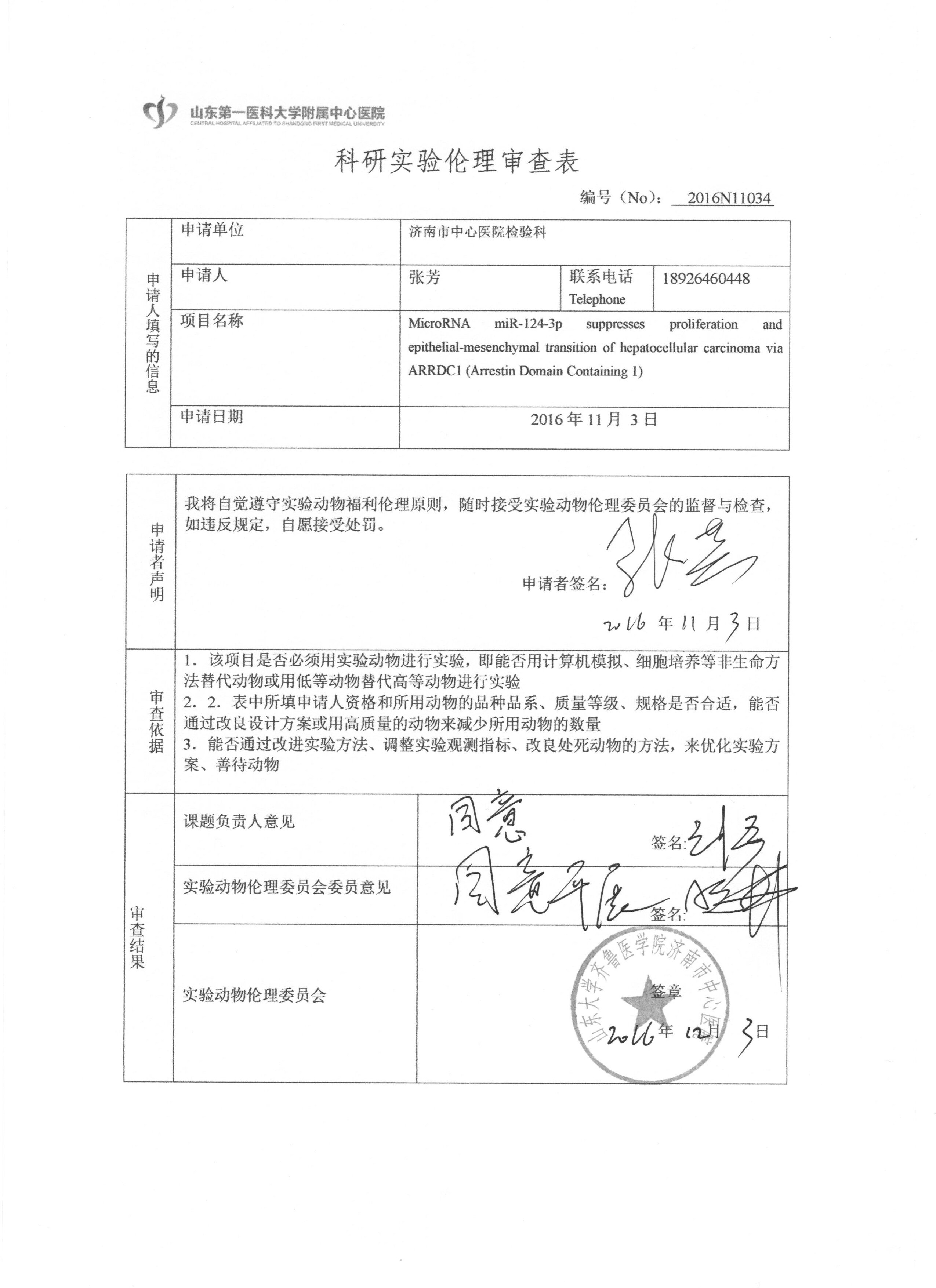

Supplement: Supplemental Material [file KBIE_A_2051686_SM2137.jpg]
